# Myeloid sarcoma presenting as intestinal obstruction: A case report of the first presentation of acute myeloid leukemia

**DOI:** 10.1016/j.ijscr.2025.111522

**Published:** 2025-06-14

**Authors:** Deepsikha Dharamsaktu, Anuradha Pandit, Charanjeet Ahluwalia, Sana Ahuja

**Affiliations:** Department of Pathology, Vardhman Mahavir Medical College and Safdarjung Hospital, New Delhi, India

**Keywords:** Myeloid sarcoma, Pediatric AML-M5, Intestinal obstruction, c-KIT mutation

## Abstract

**Introduction and importance:**

Myeloid sarcoma (MS), a rare extramedullary tumor composed of immature myeloid cells, most commonly affects the skin, lymph nodes, and gastrointestinal tract. Rarely, it presents in isolation or in pediatric patients. Misdiagnosis is frequent due to its morphological similarity to lymphomas and lack of clinical context. Early identification is crucial as MS often precedes or accompanies acute myeloid leukemia (AML) and requires immediate treatment.

**Case presentation:**

A two-and-a-half-year-old female presented with fever, oral ulcers, abdominal pain, and bleeding per rectum. Imaging revealed ileo-ileal intussusception, necessitating emergency surgery. Intraoperatively, a large lymph node and gangrenous bowel segment were resected. Histopathology showed diffuse infiltration by atypical mononuclear cells. Immunohistochemistry revealed positivity for LCA, MPO, CD13, CD33, BCL2, and CD56. A diagnosis of MS was confirmed. Subsequent hematological evaluation revealed AML-M5 with 90 % blasts, and a c-KIT exon 17 mutation. The patient responded well to induction chemotherapy (COG protocol) and remains in remission on consolidation therapy.

**Clinical discussion:**

Gastrointestinal MS is uncommon, especially in pediatric patients. AML-M5 with monocytic differentiation and CD56 expression is associated with increased risk of extramedullary involvement. The presence of c-KIT mutation may indicate aggressive disease and poor prognosis. Accurate diagnosis requires immunohistochemistry and molecular studies.

**Conclusion:**

This case highlights the importance of considering MS in pediatric patients with intestinal masses. Timely diagnosis using a multimodal approach enables prompt initiation of therapy and may improve outcomes.

## Highlights

• Myeloid sarcoma can mimic common pediatric abdominal emergencies like intussusception.
• Immunohistochemistry and molecular profiling are essential for accurate diagnosis.
• Early detection of extramedullary disease enables timely AML-directed therapy.

## 1 Introduction

Myeloid sarcoma (MS), also known as granulocytic sarcoma or chloroma, is a rare tumor composed of myeloid blasts occurring at extramedullary sites. Commonly affected areas include skin, lymph nodes, and gastrointestinal tract, while involvement of lungs, brain, and reproductive organs is less frequent [1,2]. MS can occur in various clinical contexts: as a presentation of AML, during relapse, in association with myeloproliferative neoplasms (MPNs) or myelodysplastic syndromes (MDS), or following cytotoxic therapy. All these are termed as secondary MS. Rarely, it can presents as a de novo lesion, known as primary MS, or as isolated MS when bone marrow appears morphologically normal [[1], [2], [3]].

Myeloid sarcoma is a rare disease presents as a tumor like mass lesion involving any anatomical site except bone marrow, which is composed of myeloid blasts with or without maturation effacing the architecture of the affected site, most commonly skin, lymph nodes, and gastrointestinal tract and rarely reproductive organs, lung and brain [1].

Myeloid sarcoma presents in patients with diagnosed cases of AML, patients undergoing allogenic stem cell transformation, as relapse of AML, diagnosed case of MPN and MDS or secondary to cytotoxic therapy. These all scenarios are secondary myeloid sarcomas; however, myeloid sarcoma can occur “De novo” with no pre-existing diagnosis termed as primary myeloid sarcoma. Myeloid sarcoma with morphologically normal bone marrow is known as isolated myeloid sarcoma and is very rare 1.4–9 % [[1], [2], [3]].

The myeloid sarcoma is known to affect males 2 times more than females with a median age group of 46 to 59 years [2,4].

Here we report a case of myeloid sarcoma with first presentation as intestinal obstruction in a two-and-a-half-year-old female child reported in line with SCARE guidelines [5].

## 2 Case presentation

A two-and-a-half-year-old female presented to the emergency department with a 3-day history of fever, oral ulcers, abdominal pain, and bleeding per rectum, accompanied by an inability to pass stool.

On examination, the patient appeared ill and febrile (temperature: 38.5 °C), with a heart rate of 130 bpm and blood pressure of 90/60 mmHg. Abdominal examination revealed distension and tenderness in the lower abdomen without palpable masses.

Laboratory investigations revealed hemoglobin 8.5 g/dL, total leukocyte count 15,000/μL, platelet count 90,000/μL, C-reactive protein 25 mg/L, and lactate dehydrogenase 600 U/L. Abdominal ultrasonography revealed an ileo-ileal intussusception. An abdominal X-ray showed dilated bowel loops with multiple air-fluid levels.

Given the clinical presentation and imaging findings suggestive of bowel obstruction due to intussusception, an emergency laparotomy was performed.

Intraoperatively, an ileo-ileal intussusception was identified, with a 16 cm segment of the small intestine appearing gangrenous. A 4 × 3 cm mesenteric lymph node was also noted. A resection of the affected bowel segment was performed, followed by the creation of a double-barrel ileostomy. The resected bowel segment and the lymph node were sent for histopathological examination.

Gross examination of the bowel segment revealed a 5 cm area of gangrenous thickening. The lymph node measured 1 cm in diameter.

Histopathological examination revealed effacement of the bowel wall architecture due to diffuse infiltration of singly scattered atypical cells that invaded the mucosa, submucosa, and muscularis propria. These cells were intermediate to large, with scant to moderate eosinophilic cytoplasm, round to oval nuclei with irregular nuclear membranes, open chromatin, and 0–1 prominent nucleoli. The lymph node was also diffusely infiltrated by similar atypical cells, effacing the nodal architecture.

**Fig. 1:**
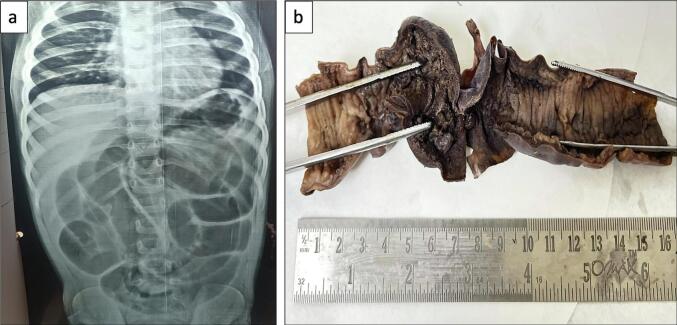
Radiological and gross images. a) X-ray whole abdomen showing dilated bowel loops. b) Gross specimen of small intestinal segment showing an ulcerated area and a thinned out bowel wall.

**Fig. 2:**
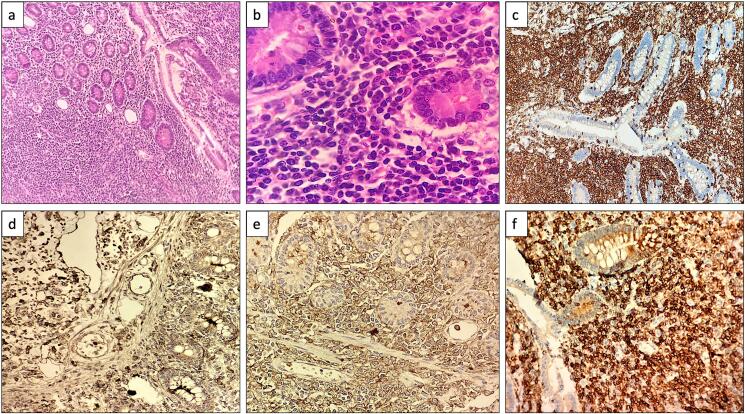
Histopathological and immunohistochemical images. a, b) Hematoxylin and eosin stained sections showing effacement of small intestine architecture with atypical cells [100×, 200×] c–f) Immunohistochemistry showing positive immunostaining for LCA (c), MPO (d), CD13 (e) and CD33 (f) [200×].

Immunohistochemistry demonstrated that these cells were positive for leucocyte common antigen (LCA), myeloperoxidase (MPO), CD13, CD33, BCL2, and CD56, and negative for TdT, CD34, BCL6, CD23, and CD10. The Ki-67 proliferation index was 80–90 % (Fig. 1, Fig. 2).

These histopathological and immunohistochemical findings confirmed the diagnosis of myeloid sarcoma.

Postoperatively, the patient's condition stabilized; however, she developed overt leukocytosis, anemia, and thrombocytopenia. Peripheral smear examination revealed 68 % blasts, which were 2–2.5 times the size of small lymphocytes, with high nucleocytoplasmic ratios and scant cytoplasm.

Bone marrow aspiration and flow cytometry confirmed AML-M5 with 90 % blasts/promonocytes. Flow cytometry showed aberrant expression of CD13, CD33, CD64, and CD123, and absence of CD34, CD14, CD117, and CD56. The immunophenotypic profile supported AML with monocytic differentiation. Molecular analysis revealed a c-KIT exon 17 mutation.

The patient was initiated on induction chemotherapy following the Children's Oncology Group (COG) protocol for pediatric AML, using the ADE regimen—cytarabine, daunorubicin, and etoposide. Supportive care included packed red cell and platelet transfusions, empirical antibiotics, antifungals, and tumor lysis prophylaxis. Following the induction phase, hematologic remission was achieved, and the patient was continued on consolidation chemotherapy with high-dose cytarabine. The patient remains under close follow-up for treatment response, complications, and minimal residual disease (MRD) assessment.

## 3 Discussion

This case highlights a rare presentation of myeloid sarcoma in a pediatric patient, initially manifesting as intestinal obstruction due to ileo-ileal intussusception. Gastrointestinal MS accounts for approximately 10 % of MS cases, with the small intestine being an uncommon site [[6], [7], [8]]. Literature review showed many cases with similar presentation (abdominal pain, nausea, vomiting, SAIO and IO) as this case; however, all the reported cases are of adult patients (Table 1) [[9], [10], [11], [12], [13], [14], [15]]. This case is of a 2 and half year old child, which is a very rare age for myeloid sarcoma.

**Table 1:** Comparison of previously reported cases on small intestinal myeloid sarcoma.

	Author	Age/sex	Presentation	Radiological findings	Site of MS	Progression
1	Cicilet et al. [9]	45/F	Severe lower abdominal pain and vomiting, tenderness in the suprapubic region	CT scan: Eccentric focal bowel wall thickening involving the ileum with few discrete enlarged perifocal lymph nodes	Terminal ileum	Aleukemic leukemia
2	Minagi et al. [8]	33/F	Recurrent abdominal pain.	CT scan: Thick ileal mass	Ileum	Bone marrow negative
3	Thingujam et al. [10]	38/M	Intermittent, upper, vague abdominal pain for 1 month	Radiological diagnosis: GIST/NHL	Mid ileum	Isolated MS in non-leukemic patients
		31/M	Abdominal distension, altered bowel habits, and a palpable abdominal mass	Radiological diagnosis: GIST	Terminal ileum	Isolated MS in non-leukemic patients
		26/M	Abdominal distension, altered bowel habits, and a palpable abdominal mass	Radiological diagnosis: Tubercular	Terminal ileum	Isolated MS in non-leukemic patients
4	Yoldaş et al. [11]	44/M	Abdominal pain, distention, nausea, and vomiting × 5 months	CT scan: wall thickening at the level of the ileal loops compatible with submucosal hematoma	Ileum	Isolated MS
5	Abou-Ghanem et al. [12]	32/F	Abdominal pain and vomiting for 3 days.	CT scan: small bowel obstruction with mass arising in the distal ileum, simulating carcinoid tumors	Ileum	Isolated MS
6	Yoshida et al. [13]	47/M	Abdominal pain, nausea, emesis and diarrhea for four days.	CT scan: high-grade SBO with asymmetric irregular bowel wall thickening of distal ileum	Ileum	Isolated MS
7	Nikolovski et al. [14]	22/M	Abdominal pain and vomiting with a palpable mass in the right medial abdominal quadrant	CT scan: Mass originating from the ileal mesentery and a part of the ileum with ileal wall thickening	ileum	Bone marrow WNL
8	Girelli et al. [15]	64/F	Chronic diarrhea	Abdominal CT: stricture of the distal ileum	Ileum	AML FAB M1
9	Wang et al. [22]	25	Intermittent pain × 6 months	CT scan: concentric wall thickening of the jejunum	Jejunum with mesenteric lymph node	Progress to AML in 3 months with simultaneously involved the small intestine, 10kidneys, mesentery, and mesenteric lymph nodes
10	He et al. [23]	40/F	Upper vague abdominal pain for 1 month	CT showed a soft tissue density mass in the mesenteric region and a wall thickening of the jejunum.	Jejunum	Primary or isolated MS
11	Mizumoto et al. [6]	54/M	Abdominal pain and vomiting	CT scan: Thickening of the jejunal wall was still causing obstruction	Jejunum	Aleukemic leukemia
12	Barnard et al. [16]	49/M	Progressive obstructive gastrointestinal symptoms	CT scan: Segmental thickening and high-grade stenosis of mid and distal ileum, with associated mesenteric lymphadenopathy	Mid-jejunum	AML
13	Antic et al. [24]	28/F	Abdominal pains and jaundice	Esophagogastroduodenoscopy (EGDS) and rectoscopy indicated infiltrative changes to the stomach, duodenum and rectum	Duodenum	Bone marrow: AML
14	Kim et al. [17]	49/M	Recurrent central colicky abdominal pain	Mass-like lesion involving jejunum	Jejunum	Non-leukemic myeloid sarcoma
15	McCusker et al. [25]	22/F	Presented as Acute Appendicitis underwent an appendectomy. Postoperatively, she continued to be symptomatic	Diagnostic laparoscopy: Multiple small bowel masses with diffuse abdominal and pelvic lymphadenopathy	Small intestine and mesenteric lymph node	AML
16	Kheirkhah et al. [6]	60/F	Patient complained of persistent abdominal pain, with coffee-ground emesis and one episode of melena	CT scan: showed thickening of the gastric wall near the gastroesophageal junction with multiple enlarged lymph nodes.	Gastric Fundus and proximal duodenum	AML
17	Yu et al. [18]	31	Upper abdominal pain, melena, vomiting and jaundice	CT scan: mass in gastric antrum area and infiltration into duodenum, gallbladder and head of the pancreas, with possible retroperitoneal lymph node metastasis	Multiple organ gastric antrum, duodenum, retroperitoneal lymph node, breast and B/L orbits	Disease relapsed at 5 months

Intestinal myeloid sarcoma is a rare entity. The radiological diagnosis in most of the reviewed cases was gastrointestinal stromal tumor, lymphoma, tubercular etiology, metastatic carcinomas [[8], [9], [10], [11],[16], [17], [18]]. Our case on USG was seen as an intussusception.

In this case patient later developed AML-M5, a rare entity comprising only 2–8 % of AML cases [8,19]. Literature review showed many cases with AML; however, only few described FAB Classification. Myeloid sarcoma is more commonly associated with AML FAB M2, M4 and M5. Expression of Surface CD56, CD11b, and monocytic differentiation is reported to be associated with increased risk of extramedullary involvement, which are seen in this case [2,7,8,20].

A study done by Faaij et al. shows the presence of chemokine receptors CCR5, CXCR4, CXCR7 and CX3CR1 in blasts of AML in extramedullary sites, which was not present in AML blasts in bone marrow and peripheral blood [20].

A study done by Wang et al. described a highly invasive monocytic leukemic blast line SH-1, It shows strong expression of matrix metalloproteinase 2 (MMP2), Membrane type 1 MMP (MT1-MMP) and tissue inhibitor of metalloproteinase 2 (TIMP-2) [21].

The most common molecular aberration in abdominal myeloid sarcoma cases is of fusion protein CBFB-MYH11 [7]. However, A retrospective study done by Zhao H et al. in 118 patients of myeloid sarcoma cited KIT as one of the most common genetic mutations (16.6 %) [4]. This case also demonstrates mutation of c-KIT (exon 17), which is seen affecting 15 % of the myeloid sarcoma cases. C-kit is a tyrosine kinase receptor and an exon 17 mutation in c-kit leads to disruption of the normal self-inhibition loop. Leading to unchecked sustained activation of downstream signalling pathways including Pi3k/AKT, JAK/STAT and MAPK. This activation promotes cell proliferation, migration and survival. c-KIT (exon 17) mutation is also responsible for imatinib resistance, hence, it can be associated with poor prognosis.

Misdiagnosis of myeloid sarcoma is common and can be attributed to several factors, including its morphological resemblance to lymphomas and other small round blue cell tumors [4,12], the unavailability of clinical history and hematological findings at the time of biopsy interpretation, and the aberrant expression of lymphoid markers such as CD3 and CD79a in some myeloid sarcoma cases [7,8]. Additionally, cases of primary or isolated myeloid sarcoma often pose a diagnostic challenge due to the absence of concurrent bone marrow involvement or systemic disease [6]. To reduce the risk of misdiagnosis, it is recommended to include myeloid markers such as MPO and CD117 in the immunohistochemical panel used for lymphoma workup.

Differential diagnoses of myeloid sarcoma include Lymphoblastic lymphoma, Burkitt lymphoma, DLBCL, Blastoid variant of mantle cell lymphomas, Mature T cell neoplasm with primary or secondary GI tract involvement, Myeloid metaplasia (multilineage hematopoiesis with a progressive stage of maturation), metastatic malignancies [8]. melanomas, undifferentiated carcinomas [7,21].

While MS itself is not an independent prognostic factor, it often indicates aggressive disease. Isolated MS frequently progresses to overt AML within months [7]. A retrospective study done by Zhao H et al. in 118 patients of myeloid sarcoma show Medial survival time was 4 months, ranging from1 to 51 months. The median survival time of patients who received allogenic Hematopoietic stem cell transplantation was 19 months, ranging from 8 to 51 months [4].

Therapeutic approaches in myeloid sarcomas are tailored according to genetic abnormality, systemic chemotherapy and stem cell transplantation [7].

## 4 Conclusion

This case illustrates the diagnostic complexity of myeloid sarcoma in a pediatric AML-M5 patient with intestinal involvement. Accurate diagnosis relies on integrated clinical, histological, immunophenotypic, and molecular analysis. Early recognition of extramedullary disease is vital for the timely initiation of appropriate therapy.

## CRediT authorship contribution statement

DD, AP, CA were responsible for the reporting and diagnosis of the case. DD and AP were major contributors to the writing of the manuscript while CA, SA were responsible for the article review and editing. All authors read and approved the final manuscript.

## Consent for publication

Written informed consent was obtained from the patient guardian for publication of this case report and accompanying images. A copy of the written consent is available for review by the Editor- in -Chief of this journal on request.

## Ethical approval

This case study was conducted in accordance with the Declaration of Helsinki.

## Previous presentation/publication

None.

## Guarantor

SA.

## Funding

This research received no funding.

## Declaration of competing interest

All authors declare that there is no conflict of interest regarding the publication of this report.

## Data Availability

Supporting data will be available upon reasonable request.
